# A Digital Health Intervention for Concussion: Development and Clinical Feasibility Study

**DOI:** 10.2196/43557

**Published:** 2023-02-01

**Authors:** Christine d'Offay, Xin Yi Ng, Laura Alexander, Alison Grant, Julia Grahamslaw, Claudia Pagliari, Matthew J Reed, Alan Carson, David C Gillespie, Aimun A B Jamjoom

**Affiliations:** 1 Centre for Population Health Sciences Usher Institute The University of Edinburgh Edinburgh United Kingdom; 2 HeadOn Health Ltd Edinburgh United Kingdom; 3 University of Edinburgh Medical School The University of Edinburgh Edinburgh United Kingdom; 4 Centre for Clinical Brain Sciences The University of Edinburgh Edinburgh United Kingdom; 5 The Emergency Medicine Research Group Edinburgh (EMERGE) Royal Infirmary of Edinburgh Edinburgh United Kingdom; 6 Acute Care Edinburgh Usher Institute The University of Edinburgh Edinburgh United Kingdom; 7 Department of Clinical Neuroscience Edinburgh Royal Infirmary Edinburgh United Kingdom

**Keywords:** concussion, digital intervention, behavior change, feasibility study

## Abstract

**Background:**

Concussion is a common condition that can lead to a constellation of symptoms that affect quality of life, social integration, and return to work. There are several evidence-based behavioral and psychological interventions that have been found to improve postconcussion symptom burden. However, these are not routinely delivered, and individuals receive limited support during their concussion recovery.

**Objective:**

This study aimed to develop and test the feasibility of a digital health intervention using a systematic evidence-, theory-, and person-based approach.

**Methods:**

This was a mixed methodology study involving a scoping review (n=21), behavioral analysis, and logic model to inform the intervention design and content. During development, the intervention was optimized with feedback from individuals who had experienced concussions (n=12) and health care professionals (n=11). The intervention was then offered to patients presenting to the emergency department with a concussion (n=50). Participants used the intervention freely and input symptom data as part of the program. A number of outcome measures were obtained, including participant engagement with the intervention, postconcussion symptom burden, and attitudes toward the intervention. A selection of participants (n=15) took part in in-depth qualitative interviews to understand their attitudes toward the intervention and how to improve it.

**Results:**

Engagement with the intervention functionality was 90% (45/50) for the symptom diary, 62% (31/50) for sleep time setting, 56% (28/50) for the alcohol tracker, 48% (24/50) for exercise day setting, 34% (17/50) for the thought diary, and 32% (16/50) for the goal setter. Metrics indicated high levels of early engagement that trailed off throughout the course of the intervention, with an average daily completion rate of the symptom diary of 28.23% (494/1750). A quarter of the study participants (13/50, 26%) were classified as *high engagers* who interacted with all the functionalities within the intervention. Quantitative and qualitative feedback indicated a high level of usability and positive perception of the intervention. Daily symptom diaries (n=494) demonstrated a wide variation in individual participant symptom burden but a decline in average burden over time. For participants with Rivermead scores on completion of HeadOn, there was a strong positive correlation (*r*=0.86; *P*<.001) between their average daily HeadOn symptom diary score and their end-of-program Rivermead score. Insights from the interviews were then fed back into development to optimize the intervention and facilitate engagement.

**Conclusions:**

Using this systematic approach, we developed a digital health intervention for individuals who have experienced a concussion that is designed to facilitate positive behavior change. Symptom data input as part of the intervention provided insights into postconcussion symptom burden and recovery trajectories.

**Trial Registration:**

ClinicalTrials.gov NCT05069948; https://clinicaltrials.gov/ct2/show/NCT05069948

## Introduction

Concussion is a common condition that can occur in a range of circumstances, including falls, assaults, road traffic collisions, and playing sports [1]. Estimates place the incidence of concussion at 600 per 100,000, and it is a major cause of presentation to the emergency department (ED), with approximately 2 million visits in the United States per year [1,2]. Individuals can experience a constellation of postconcussion symptoms, including physical (headaches and dizziness), cognitive (difficulty concentrating and memory problems), and emotional (depression and anxiety) symptoms as well as sleep disturbances [3]. Although these symptoms typically improve over time, a considerable proportion of individuals remain with persistent symptoms up to a year after their injury [4,5]. Postconcussion symptom burden is associated with poorer health-related quality of life [6], reduced community integration [7], work absenteeism [8], and increased use of health care resources [9]. Despite the serious public health concern that concussion poses, individuals are typically offered limited support and follow-up after their injury [10,11]. This is a concern as several behavioral and psychological interventions have been found to improve postconcussion symptom burden, including early educational material [12], cognitive behavioral therapy (CBT) [13], and aerobic exercise [14]. Importantly, intervening early in the natural history following concussion appears to play a role in improving outcomes [12,15]. Digital health interventions are increasingly being used to manage a range of medical conditions [16,17]. They are scalable and can be cost-effectively delivered early in the clinical course of the disease. A review of digital solutions for concussion found limited options and a lacking evidence-base [18]. To address this problem, we developed HeadOn—a digital health intervention that uses behavior change techniques to encourage positive behaviors to facilitate postconcussion recovery. The intervention was developed using a systematic evidence-, theory-, and person-based approach and the Medical Research Council (MRC) guidance on the development of complex interventions [19]. In this paper, we describe the development of the intervention and subsequent clinical feasibility study examining the acceptability and use of the intervention by a cohort of participants presenting to an ED with a concussion.

## Methods

### Scoping Review

A literature search was conducted in February 2021 to identify relevant studies. The electronic databases used were MEDLINE (via Ovid), Scopus, PsycINFO (via Ovid), and the Cochrane Library. The following terms and their derivatives were used in the electronic search: “concussion,” “post-concussion syndrome,” “mild traumatic brain injury,” “cognitive behavioral therapy,” “exercise therapy,” “self-management,” “self-care,” and “health education” (Multimedia Appendix 1 [20-40]). The inclusion criteria were as follows: (1) peer-reviewed English-language articles published from the year 2000 onward, (2) qualitative research (interviews, surveys, and focus groups), and (3) studies examining behavioral (exercise and physical rehabilitation, rest, and self-management) and psychological (health education, CBT, counseling, psychotherapy, and cognitive rehabilitation) interventions for concussion (including postconcussion syndrome). After removing duplicates, the titles and abstracts of papers that met the inclusion criteria were screened by 2 reviewers (XN and AABJ), and full-text article reviews were carried out on eligible papers. The bibliographies of relevant articles found during screening were also searched manually. The included articles were reviewed, and data were extracted and organized into a predefined table of potential barriers to and facilitators of the use of interventions. These were mapped onto the following domains: intervention engagement, information, symptom monitoring, lifestyle and behavior, thoughts and emotions, design, and technical aspects. The PRISMA (Preferred Reporting Items for Systematic Reviews and Meta-Analyses) scoping review checklist is detailed in Multimedia Appendix 1.

### Behavioral Analysis

The behavioral analysis involved linking the barriers identified in the scoping review to target behaviors (intervention engagement, symptom monitoring, healthy lifestyle habits, addressing negative thoughts, and goal setting). The Behavior Change Wheel (BCW) [41] and taxonomy of behavior change techniques [42] were used as the theoretical framework to systematically address each barrier. The BCW was used to identify the target construct and intervention functions, followed by the taxonomy to identify the appropriate behavior change technique to address the barrier. This ensured a systematic and theoretically driven approach to addressing barriers to behavior change.

### Logic Model

A logic model was developed based on the outcome of the scoping review and behavioral analysis. A logic model is a graphic representation of the shared relationships among the activities, outputs, and outcomes of a program. For HeadOn, the model included five parts: (1) the clinical problem that HeadOn aims to address; (2) the intervention targets, which are the behaviors that HeadOn aims to promote; (3) the intervention ingredients, which are the program components of HeadOn; (4) examined mechanisms, which are the metrics to measure the impact of HeadOn; and (5) clinical outcomes to examine the efficacy of HeadOn.

### Optimization Study 1: Concept and Design Review

Once intervention planning was complete, the HeadOn intervention components were defined, and the user interface was designed. Feedback was sought at this stage with face-to-face questionnaire-based surveys conducted with National Health Service patients presenting to the ED at the Royal Infirmary of Edinburgh and with a range of health care professionals who are involved in the management of concussions. The intervention concept was explained to the patients and health care professionals, and they were shown figures of the proposed design. The interviewer (LA) went through a structured questionnaire (Multimedia Appendix 1). Each interview lasted approximately 15 minutes.

### Optimization Study 2: Prototype Assessment

Having completed a prototype of HeadOn, a series of “think-aloud” interviews were conducted with volunteers who had experienced a concussion. Volunteers were recruited from concussion support groups on a social media site and via advertising through a brain injury charity and were invited to participate in the interview, which was conducted by videoconference. During the interviews, participants were given access to the intervention and were asked a series of questions (conducted by CD) about the usability and initial perception of the intervention. Participant comments were recorded, and areas of feedback were implemented.

### Mixed Methods Clinical Feasibility Study

A prospective mixed methods study was conducted in which participants who had experienced a concussion were given access to HeadOn. This study included both quantitative and qualitative components. The main quantitative study examined participant engagement with HeadOn and a range of clinical outcomes. Participants could also volunteer to take part in a qualitative substudy involving an in-depth interview about their experience with HeadOn. The study ran for a predefined 6-month period between November 2021 and April 2022. Patients presenting to the Edinburgh Royal Infirmary and St John’s Hospital EDs with a concussion were invited to participate. Inclusion criteria were patients aged ≥16 years presenting with a concussion, which was defined according to the American Congress of Rehabilitation Medicine—a traumatically induced disruption of brain function presenting as any alteration of mental status, loss of consciousness, or posttraumatic amnesia [43]. Participants were required to have a Glasgow Coma Scale score of 13 to 15 on initial presentation to the ED and would need to be able to register with HeadOn within 14 days of their concussion. Exclusion criteria were patients requiring surgical management of their cranial injury, substantial other associated injuries requiring hospitalization (spinal injury; fractures; and abdominal, cardiothoracic, or vascular injuries), lack of capacity to provide consent, non-English speakers, and patients in police custody or in prison. To take part in the study, participants gave written informed consent through an electronic consent system. Consent was obtained either while the patient was in the ED or after discharge up to 14 days after their injury. Following recruitment, study participants were taken through the HeadOn registration process, which included an introductory video and the completion of the Rivermead Post-Concussion Symptom Questionnaire [44] and Patient Health Questionnaire-9 (PHQ-9) [45]. In addition, a series of researcher-led anonymized data points was collected, including demographics, date of concussion, and neurological and imaging findings. Participants were then given open access to HeadOn, which ran over a 5-week period. At the completion of HeadOn, participants were invited to complete the Rivermead Post-Concussion Symptom Questionnaire and PHQ-9 again. Participants were also invited to complete the mHealth App Usability Questionnaire (MAUQ) to provide feedback on the usability of HeadOn [46]. As HeadOn did not provide functionality to communicate with a health care provider, the final 4 questions of the MAUQ that focused on this were excluded. There was no control group in this feasibility study. Flow diagrams of the study design and recruitment can be found in Figures S1 and S2 in Multimedia Appendix 1. The study protocol was published on ClinicalTrials.gov (NCT05069948) on October 6, 2021. The CONSORT (Consolidated Standards of Reporting Trials) 2010 checklist of information to include when reporting a pilot or feasibility trial is reported in Multimedia Appendix 1 [47].

### Qualitative Interviews

As part of the consent process for the mixed methodology study, participants could opt in to take part in the qualitative interviews. Study participants who consented to take part in the qualitative interviews (44/50, 88%) were contacted by email 3 weeks after registration with HeadOn to arrange an interview date. Participants who did not initially respond were contacted once more to organize a qualitative interview. Those who did not respond after 2 attempts were sent a short electronic survey about their attitudes toward HeadOn (no responses were gathered through this method). Interviews were conducted by 1 interviewer (CD—female Master of Science by Research student) via telephone and recorded using an encrypted recording device. The interviews were conducted directly between CD and the interviewee without anyone else participating in the conversation. Average interview time was between 15 and 45 minutes. CD had previous experience in conducting qualitative interviews and had completed additional training in conducting and analyzing qualitative research. The interview schedule can be found in Multimedia Appendix 1. Owing to CD’s close involvement in the design and development of the intervention, careful attention was paid to designing the interview guide to reduce the risk of bias. In addition, it was made clear that CD was interested in the participants’ experience of using (or not using) the intervention, of which they were the experts. The recordings were transcribed by CD within 24 hours. Transcribed interviews were then coded and analyzed using NVivo (version 12; QSR International). Deductive thematic analysis was used to identify barriers to and facilitators of using HeadOn, which were subsequently mapped onto the same domains that were used in the scoping review: patient engagement, information, symptom tracking, lifestyle and behavior, thoughts and emotions, design, and technical aspects. To check for consistency, a second researcher (AABJ) reviewed a random selection (3/15, 20%) of the transcripts and conducted thematic analysis. No significant differences were found between the analyses. The interviews were conducted until data saturation was achieved. Data saturation indicates that, based on the data that have been collected and analyzed so far, no further data collection and analysis is necessary to gain new insights. Our stopping criterion for data saturation was the emergence of no new themes in 3 consecutive interviews having conducted a minimum of 8 interviews. To ensure that no new themes emerged, the first 8 interviews were analyzed and coded. Following this, each new interview was coded and analyzed sequentially until 3 consecutive interviews found no new themes. Data saturation was achieved at 15 interviews and was agreed upon by 2 researchers (CD and AABJ). The COREQ (Consolidated Criteria for Reporting Qualitative Research) guidelines [48] can be found in Multimedia Appendix 1.

### Statistical Analysis

Engagement with functionality was defined as the inputting of data into a given functionality as part of the HeadOn program. A *high engager* was defined as a participant who engaged with all the functionalities of HeadOn during the 5-week program. A *nonengager* was defined as a participant who did not engage with any HeadOn functionality after initial registration. A “clinically significant” change in PHQ-9 score was defined as a change of ≥5 points from baseline [45]. The normality of continuous data was checked using the D’Agostino and Pearson tests. The unpaired Student 2-tailed *t* test was used for examining parametric data. The Fisher exact test or chi-square test was used to analyze categorical data. For correlation, the Pearson correlation coefficient was calculated. Prism (version 9.3.1; GraphPad Software) was used for the statistical analysis.

### Ethics Approval

The methods were performed in accordance with relevant guidelines and regulations and approved by the North West - Preston Research Ethics Committee (21/NW/0211) on September 14, 2021. Informed consent was provided by study participants for the clinical feasibility study. Participants did not receive any compensation as part of the study. The data captured as part of the study were kept in accordance with the University of Edinburgh and National Health Service Lothian privacy and data protection requirements. The databases used in this study met industry-standard data protection protocols. Researcher-captured data were anonymous, and the exported data from HeadOn were deidentified and encrypted before analysis.

## Results

### Development of the Intervention

#### Rapid Scoping Review

**Figure 1:**
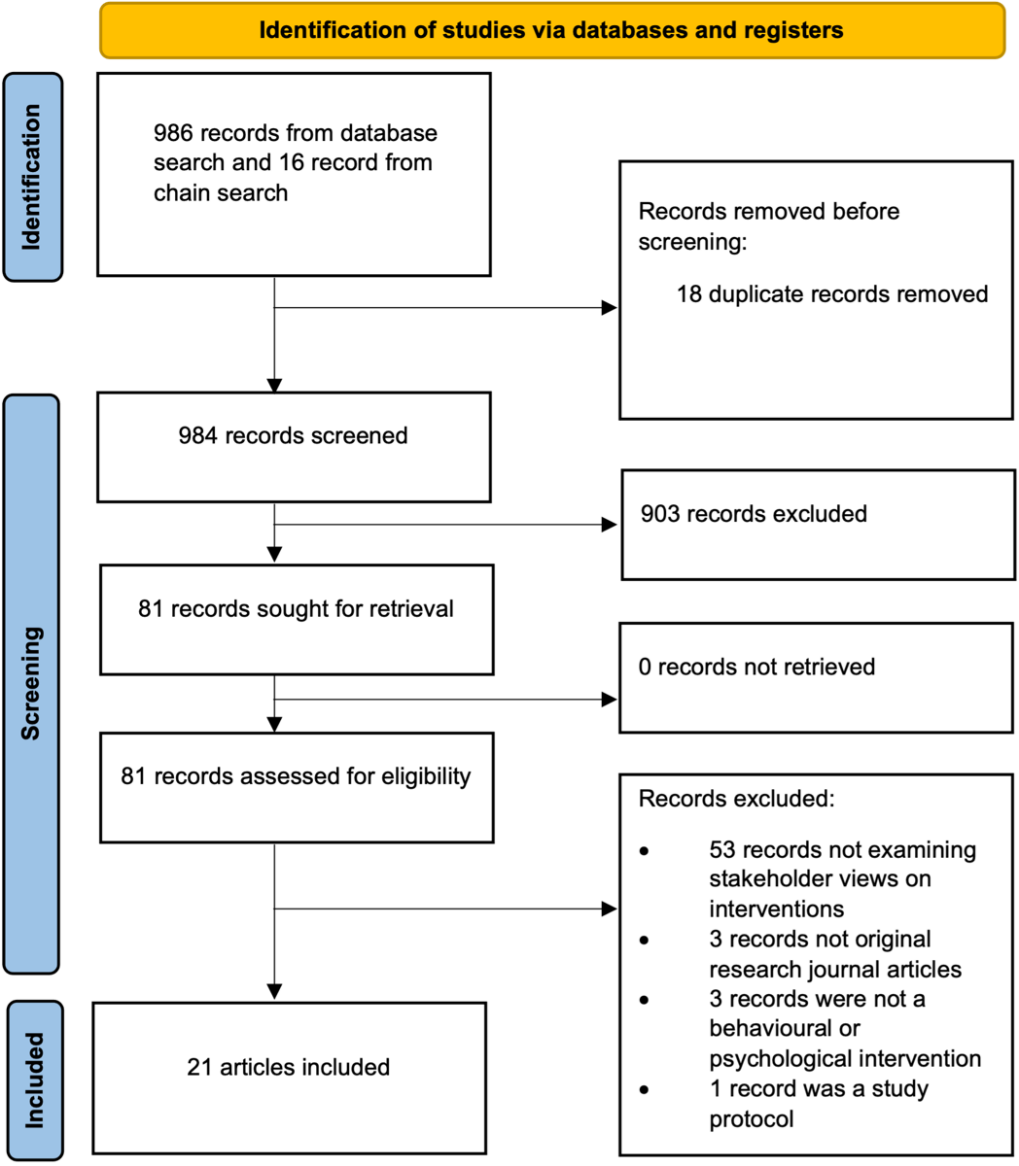
The PRISMA (Preferred Reporting Items for Systematic Reviews and Meta-Analyses) flow diagram for the scoping review.

A scoping review was conducted to better understand the factors that prevent or facilitate patient engagement with concussion interventions. A total of 21 studies were included in the rapid scoping review (Figure 1). These studies examined a range of interventions for concussions. The characteristics of the study participants or of the intervention that appeared to affect its success were mapped onto a table of facilitators and barriers across 7 domains (intervention engagement, information, symptom tracking, lifestyle and behavior, thoughts and emotions, design, and technical aspects; Table S1 in Multimedia appendix 1). These findings were used to guide the behavioral analysis and logic model.

#### Behavioral Analysis

A behavioral analysis was conducted to address several key barriers identified in the scoping review (Table S2 in Multimedia Appendix 1). The BCW [41] was the theoretical framework used for the development of the intervention. HeadOn aimed to overcome the barriers to the target behaviors (intervention engagement, symptom monitoring, healthy lifestyle habits, addressing negative thoughts, and goal setting) by using 20 behavior change techniques [42] that targeted all 6 sources of behavior (social opportunity, physical opportunity, reflective motivation, automatic motivation, physical capability, and psychological capability) and 8 intervention functions (education, training, persuasion, incentivization, enablement, environmental restructuring, restriction, and modeling). For example, forgetfulness because of the cognitive disturbance following a concussion was one of the barriers identified during the scoping review. By providing the participants with information on how to set up a routine around accessing HeadOn, both psychological capability and automatic motivation were targeted. Physical opportunity was targeted with the use of automated emails that prompted the participants to log in.

#### Logic Model

**Figure 2:**
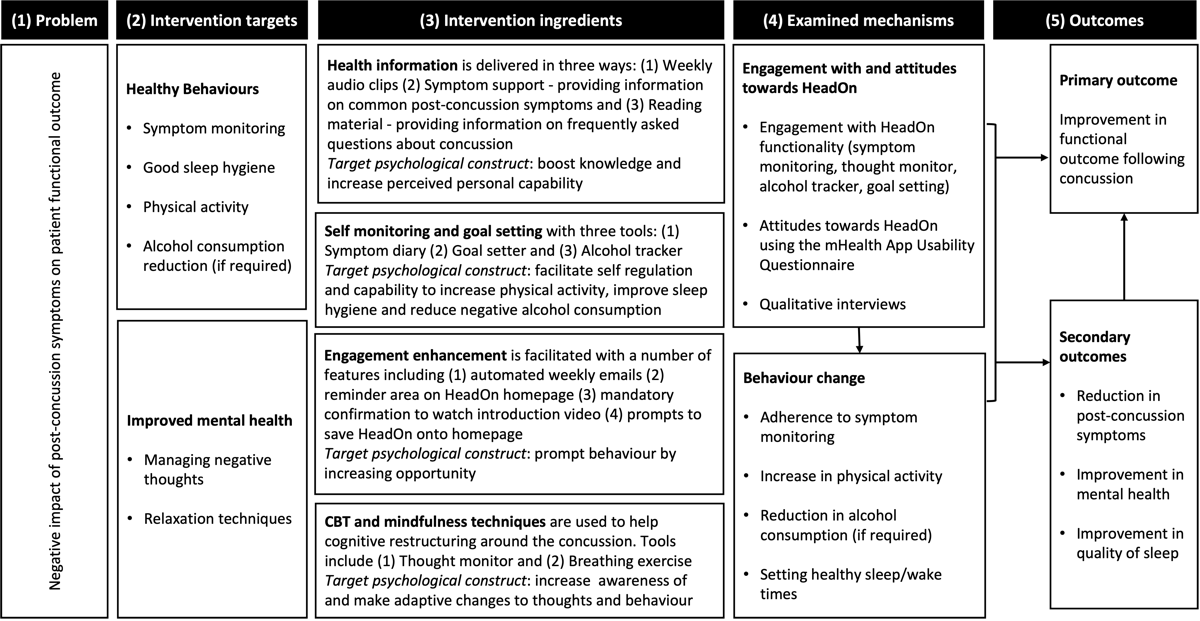
HeadOn logic model. CBT: cognitive behavioral therapy; mHealth: mobile health.

A logic model was developed to map out the mechanisms underpinning the intervention (Figure 2). This consisted of 5 parts. First, we identified the problem regarding postconcussion symptoms having a negative impact on participants’ functional outcomes. Second, we defined the intervention targets, which covered 2 areas: positive health behaviors (such as symptom monitoring, physical activity, and good sleep hygiene) and improved mental health (through CBT and relaxation techniques). Third, we laid out the intervention ingredients, which included the following: health information, self-monitoring, goal setting, engagement enhancement, and CBT and mindfulness techniques. Fourth, we defined the mechanisms that we would need to examine to determine the impact of the intervention. This included the participants’ engagement with and their attitudes toward the intervention and any associated behavior change. Finally, we laid out the outcome measures to determine the efficacy and effectiveness of the intervention. The primary outcome was an improvement in functional outcomes following concussion, and secondary outcomes included reduction in postconcussion symptoms and improvement in mental health and sleep.

#### Design of the Digital Health Intervention

The scoping review, behavioral analysis, and logic model were fed into the design of the HeadOn intervention components. HeadOn was designed to include 5 stages that run sequentially, with each stage lasting 7 days (total duration of 5 weeks). The five stages of HeadOn are (1) *Understanding your symptoms*, (2) *Sleep after a concussion*, (3) *Lifestyle and exercise*, (4) *Your thoughts*, and (5) *Getting back to baseline* (Table 1). The sequence of these stages was chosen for a number of reasons. First, we chose to examine postconcussion symptoms and sleep early as this provided the user with early knowledge of what to expect with their recovery and the type of symptoms that they may encounter. Second, we chose to have the lifestyle and exercise stage in the third week as major sporting body regulations commonly recommend that physical activity can start 2 weeks after a concussion [49]. At the start of HeadOn, an introductory video explains what HeadOn is and how to use it. At the start of each stage, an introductory audio explains the stage (accessible on the HeadOn home page), outlining important knowledge for the user and the task for that week. For each stage, the user is invited to complete a task using a piece of HeadOn functionality. All these tasks are one-off tasks except for the completion of the Symptom Diary, which the user is invited to complete every day to allow them to track their recovery. Data input into the Symptom Diary can be tracked visually through the HeadOn Progress Tracker.

**Table 1:** HeadOn intervention components.

Intervention component	Description	Task
Overview	HeadOn is a digital health intervention designed to support patients who have experienced a concussion. It contains 5 stages, each of which lasts 7 days. Each stage is introduced with an audio clip providing the patient with information about the stage and associated tasks.	Watch introductory video (one-off task)
Introduction	An introductory video explains what HeadOn is and how to use it. The patient is then invited to complete a series of questionnaires (Rivermead Post-Concussion Symptom Questionnaire and PHQ-9^a^).	Complete Rivermead questionnaire and PHQ-9 (one-off task)
Understanding your symptoms	The first week of HeadOn focuses on providing patients with information about postconcussion symptoms and techniques for managing them. This includes access to health information (including symptom-specific information and also frequently asked questions). The patient is also invited to complete a daily symptom diary and is able to view their data in the Progress Tracker area.	Complete symptom diary (recurring daily task)
Sleep after a concussion	The second week of HeadOn focuses on sleep disturbance after a concussion. The audio introduction provides the patient with important information on good sleep hygiene. The patient is also invited to set a wake-up time and bedtime for the week. They are notified by HeadOn when this time comes.	Set wake-up and sleep times (one-off task)
Lifestyle and exercise	The third week of HeadOn focuses on 2 areas: physical activity and examining alcohol consumption. All patients are invited to set 3 days of the week to perform noncontact physical activity. If the patient indicates that they had consumed alcohol at the time of the concussion, they are invited to set 3 alcohol-free days of the week and use an alcohol tracker to monitor the number of alcoholic units that they drink throughout the week.	Set exercise days (one-off task); set alcohol-free days and use alcohol tracker (one-off tasks)
Your thoughts	The fourth week of HeadOn focuses on examining the patient’s thoughts regarding their concussion. The audio introduction discusses CBT^b^ concepts regarding the role of thoughts on behavior and emotion. The patient is invited to use a thought diary to explore their thoughts regarding their concussion.	Complete the thought monitor (one-off task)
Getting back to baseline	The final week of HeadOn focuses on supporting the patient to return to their preinjury function. During this week, the patient is invited to set a goal to complete by the end of the program. They are encouraged to use the SMART^c^ approach to set and complete the goal. The patient uses the HeadOn goal-setter function, which provides reminders throughout the week.	Set goal using HeadOn goal setter (one-off task)
Completion	On completion of the HeadOn program, the patient is invited to retake the same series of questionnaires (Rivermead Post-Concussion Symptom Questionnaire and PHQ-9).	Complete Rivermead questionnaire and PHQ-9 (one-off task)

^a^PHQ-9: Patient Health Questionnaire-9.

^b^CBT: cognitive behavioral therapy.

^c^SMART: Specific, Measurable, Achievable, Relevant, and Time-Bound.

#### Optimization Study 1: Concept and Design Review

We sought stakeholder feedback by conducting a series of semistructured interviews in which the HeadOn concept (including proposed intervention components and functionality) and designs were discussed. A total of 19 interviews were conducted with 8 (42%) individuals who had presented to a local ED with a concussion and 11 (58%) health care professionals (including specialists in neuropsychology, neurosurgery, neurology, sports medicine, general practice, and physiotherapy). Across the interviewed cohort, the HeadOn concept was viewed favorably by 74% (14/19; 6/8, 75% of individuals with a concussion and 8/11, 73% of health care professionals) of the respondents, considering it a “very good-to-excellent” concept (Table S3 in Multimedia Appendix 1). Of the proposed functionalities, those focused on symptom monitoring and management (symptom diary, progress tracker, and symptom support) were viewed most favorably. The progress tracker was viewed favorably by 79% (15/19) of the respondents (6/8, 75% of individuals with a concussion and 9/11, 82% of health care professionals), the symptom support area was viewed favorably by 74% (14/19) of the respondents (5/8, 62% of individuals with a concussion and 9/11, 82% of health care professionals), and the Symptom Diary was viewed favorably by 53% (10/19) of the respondents (5/8, 62% of individuals with a concussion and 5/11, 45% of health care professionals). Over half (11/19, 58%; 4/8, 50% of individuals with a concussion and 7/11, 64% of health care professionals) of the interview respondents thought that the HeadOn design was “very good-to-excellent.” A respondent commented on the importance of using colors wisely, suggesting that warm colors were easier on the eye. Of the 8 participants who had experienced a concussion, 7 (88%) said that they would have been interested in using HeadOn if it had been available at the time. Potential barriers to the use of HeadOn that were highlighted included the need for screen time, which can be difficult for participants who have had a concussion. Having to pay to use HeadOn and a lack of evidence of its efficacy were also identified as factors that could reduce uptake and engagement.

#### Optimization Study 2: Prototype Assessment

Following the development of the intervention prototype, user testing was then conducted through “think-aloud” interviews. Think-aloud interviews provided information to understand participants’ thoughts as they interacted with HeadOn by having them think aloud while they used the intervention. A total of 4 think-aloud interviews were conducted with stakeholders. The interviews were conducted on the web using videoconference, and participants were recruited through social media support forums for concussion and via advertising through a brain injury charity. During the interviews, participants were given access to the intervention, asked a series of questions about their initial perception of the intervention, and given a series of tasks to perform. Overall, perceptions of HeadOn were positive regarding its structure and layout. Some elements of the intervention were described as difficult to locate on the application, such as a progress tracker. To address this, an explainer video was included at the start of the registration process providing information on the layout of the intervention. In conjunction, a reminder for the user to review their progress within the task section of HeadOn that linked through to the progress tracker was included. Another piece of feedback was that it was not obvious to the user that they had to listen to the audio clip at the start of each week. We addressed this in 2 ways: through the introduction of an explainer video and by redesigning the home page to ensure that the audio clip was a central feature and visually obvious to the user. One user felt strongly that using smiley faces for the Likert scale in the symptom diary was inappropriately childish, so these were changed to a numerical scale (0-4). Another user noted the lack of “back buttons” in the application, which made navigation difficult. These were also added to improve the user experience.

### Mixed Methods Clinical Feasibility Study

#### Study Overview

A total of 50 participants presenting to the ED with a concussion were recruited for the study (Table 2). The cohort had an average age of 41.9 (SD 16) years, and 54% (27/50) were female. The most common mechanism of injury was a fall (26/50, 52%), and 42% (21/50) of the participants had consumed alcohol around the time of injury. Loss of consciousness at the time of the concussion was reported by 50% (25/50) of the participants. A total of 28 participants had a brain computer tomography scan, of whom 9 (32%) were found to have a radiological abnormality. These findings included 33% (3/9) cerebral contusions, 33% (3/9) skull fractures, 22% (2/9) traumatic subarachnoid hemorrhages, and 11% (1/9) extradural hematomas. Of the 50 participants, 5 (10%) were admitted to the hospital for a median of 1 (range 1-6) day. At registration with HeadOn, the average Rivermead Post-Concussion Questionnaire score was 31 (SD 13), and the average PHQ-9 score was 13 (SD 7). There were no reported harms during the feasibility study.

**Table 2:** Participant characteristics in the clinical feasibility study (N=50).

Characteristics		Values
Age (years), mean (SD; range)		42 (16; 18-73)
**Sex, n (%)**		
	Female	27 (54)
	Male	23 (46)
**Employment status, n (%)**		
	Full-time employment	22 (44)
	Unemployed	18 (36)
	Unknown	7 (14)
	Part-time employment	3 (6)
**Alcohol consumption, n (%)**		
	No	29 (58)
	Yes	21 (42)
**Mechanism, n (%)**		
	Fall	26 (52)
	Assault	11 (22)
	Sports-related	10 (20)
	Road traffic collision	2 (4)
	Other	1 (2)
**Loss of consciousness** **, n (%)**		
	Yes	25 (50)
	No	14 (28)
	Unknown	11 (22)
**Presentation GCS^a^, n (%)**		
	15	42 (84)
	14	6 (12)
	13	1 (2)
	Unknown	1 (2)
Rivermead Post-Concussion Symptom Questionnaire score, mean (SD; range)		31 (13; 0-50)
PHQ-9^b^ score, mean (SD; range)		13 (7; 0-25)

^a^GCS: Glasgow Coma Score.

^b^PHQ-9: Patient Health Questionnaire-9.

#### Participant Intervention Engagement and Quantitative Feedback

**Figure 3:**
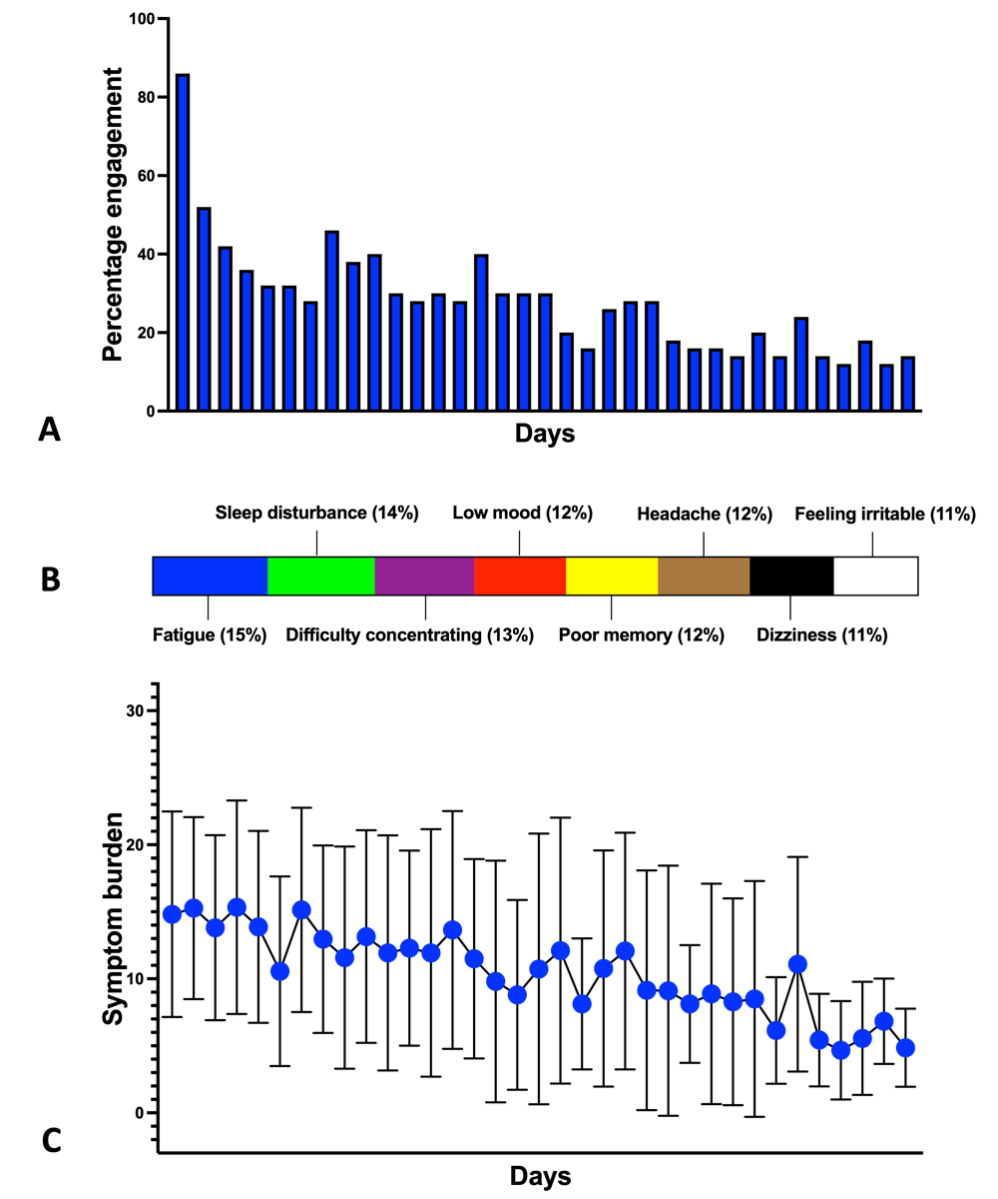
(A) The percentage of study participants completing the symptom diary. (B) Distribution of the symptom burden based on 494 symptom diary responses. (C) Temporal change in symptom burden over the course of HeadOn.

Participant engagement was quantified by examining the use of the 6 core functionality features of HeadOn. Across the 50 participants, engagement with the functionalities was 90% (45/50) for the symptom diary, 62% (31/50) for sleep time setting, 56% (28/50) for the alcohol tracker, 48% (24/50) for exercise day setting, 34% (17/50) for the thought diary, and 32% (16/50) for the goal setter. Of the 50 study participants, 13 (26%) could be classified as *high engagers* who used every function within HeadOn. Conversely, 8% (4/50) were *nonengagers* who did not use any of the functionalities after registering. *High engagers* did not differ significantly from other participants with regard to their age, sex, or Rivermead and PHQ-9 scores at registration. Study participants were invited to complete the symptom diary every day for the 5 weeks. Completion of the symptom diary started high on the first day of HeadOn (43/50, 86%) but then diminished rapidly over the course of the 5 weeks (Figure 3A). Throughout the 5 weeks, a total of 494 symptoms diaries were completed. The average daily completion rate of the symptom diary was 28.23% (494/1750). Upon completion of HeadOn, participants were invited to complete the MAUQ [46]. The questionnaire quantifies respondents’ perceptions of the usability of a mobile health app using a range from 1 to 7. A score of 7 indicates a high degree of usability. A total of 58% (29/50) of the respondents provided feedback on HeadOn using the MAUQ. From these responses, HeadOn had an average score of 6.1 (SD 1.3), indicating a high degree of usability. The MAUQ is composed of 3 domains for which HeadOn obtained the following average scores: 6.2 (SD 1.2) for Ease of Use and Satisfaction, 6.0 (SD 1.4) for System Information Arrangement, and 6.2 (SD 1.1) for Usefulness (Table S4 in Multimedia Appendix 1).

#### Qualitative Interviews

A total of 15 participants took part in the qualitative interviews. The participants had a mean age of 47 years, and 67% (10/15) were female. A comparison of the interviewed and noninterviewed cohorts revealed a statistically significantly higher percentage of high engagers in the interviewed group (*P*=.01; Table 3). The interviews were transcribed and then coded according to themes. Overall, there was a high level of satisfaction, which was consistent with the quantitative feedback from the MAUQ. Participants referred to HeadOn as “easy to use” and “straightforward.” Facilitators of and barriers to using HeadOn were identified and mapped onto the HeadOn intervention characteristics (Table 4). A major facilitator of engagement with HeadOn cited by interviewees was setting up a routine to access HeadOn by logging in at the same time each day alongside another daily activity (brushing teeth, taking medication, or going to bed), saving the app to their home page, or saving HeadOn emails to their inbox. For many participants, a high symptom burden following their injury negatively affected their physical and psychological capabilities. As a result, participants reported struggling with registration and the digital format of the intervention. In addition, some participants were advised to reduce their use of devices and screens. However, all participants were able to use the intervention successfully after the first week. Several participants who reported this difficulty at the beginning had assistance from their families or support network. Participants reported benefitting from the multiple options of either audio or text files to receive the information. Table S5 in Multimedia Appendix 1 includes the barriers and facilitators mapped onto the Capability, Opportunity, and Motivation–Behavior framework from the BCW linked to the individual interview responses. When examining how to improve HeadOn, most participants stated that they would have benefitted from an increased number of prompts and cues, such as emails, SMS text messages, and notifications. The inability to remove units from the alcohol tracker was problematic as several interviewees added accidental units that they wished to deduct. Several interviewees disliked the language used in the thought monitor. The feedback included observations that the thought monitor put too much focus on the concussion incident rather than on recovery and provided a negatively biased range of emotions (eg, anger, disgust, and anxiety) to pick from. Table S6 in Multimedia Appendix 1 contains the key qualitative interview feedback (including selected quotations) and associated changes made to HeadOn.

**Table 3:** Comparison of interviewed and noninterviewed cohorts in the feasibility study (N=50).

Characteristics		Interviewed (n=15)	Noninterviewed (n=35)	*P* value
Age (years), mean (SD)		47.2 (15.7)	39.7 (16.3)	.14
**Sex, n (%)**				.35
	Female	10 (67)	17 (49)	
	Male	5 (33)	18 (51)	
**Employment status, n (%)**				.29
	Full-time employment	7 (47)	15 (43)	
	Unemployed	7 (47)	11 (31)	
	Unknown	0 (0)	7 (20)	
	Part-time employment	1 (7)	2 (6)	
**Alcohol consumption, n (%)**				.21
	No	11 (73)	18 (51)	
	Yes	4 (27)	17 (49)	
**Mechanism, n (%)**				.30
	Fall	5 (33)	21 (60)	
	Assault	4 (27)	7 (20)	
	Sports-related	4 (27)	6 (17)	
	Road traffic collision	1 (7)	1 (3)	
	Other	1 (7)	0 (0)	
**Engagement, n (%)**				.01
	High engager	8 (53)	5 (14)	
	Non–high engager	7 (47)	30 (86)	
Rivermead Post-Concussion Symptom Questionnaire score, mean (SD)		31 (13)	31 (14)	.85
PHQ-9^a^ score, mean (SD)		14 (9)	13 (7)	.69

^a^PHQ-9: Patient Health Questionnaire-9.

**Table 4:** Barriers to and facilitators of using HeadOn based on qualitative interviews.

Intervention characteristic	Facilitators	Barriers
Intervention engagement	Family support Weekly email reminders Logging in via emails Building a routine Setting own reminder Easy to use Symptom burden Motivation to recover and belief intervention was supporting recovery Credible source Days in a row Flexibility of intervention being digital and self-directed COVID-19 pandemic improved digital literacy Being part of academic study Saved to home screen on device	Forgetfulness and memory High symptom burden after concussion Difficulty with screens and devices or digital Low symptom burden Difficulty registering Work or other commitments Not enough prompts to log in (notifications, emails, SMS text messages, and calendar reminders) Not a native app and log-in process
Information	Credible source Audio or transcript available	Difficulty with screens Difficulty concentrating
Symptom monitoring	Days in a row Ability to review progress	Repetitive Main symptom not included Progress review limited to 7 days
Exercise and lifestyle	N/A^a^	Alcohol tracker lacking ability to go back or take off units
Thought monitor	N/A	Language used did not resonate with participants or they did not feel it suited their situation Memory loss of injury Repetitive task Emotions not an issue
Technical and design	Easy to navigate Simple design Instructions Audio or text files	Registration Difficulty logging in Difficulty with screens

^a^N/A: not applicable.

#### Participant Outcomes on Completion of the Intervention

Upon completion of HeadOn, a series of participant outcomes were collected. In the cohort of 50 participants, a functional outcome using the Glasgow Outcome Scale Extended (GOSE) was collected for 58% (29/50). Of these 29 participants, 17 (59%) had a GOSE score of 8, indicating a complete functional recovery upon completion of HeadOn. A total of 44% (22/50) of the participants took the Rivermead Post-Concussion Symptom Questionnaire and PHQ-9 at completion. The average Rivermead Post-Concussion Symptom Questionnaire and PHQ-9 scores were 16 (SD 13) and 8 (SD 7), respectively. Among the 22 participants with pre- and postintervention scores, there was a statistically significant improvement in the Rivermead score upon completion of HeadOn (preintervention score mean 31, SD 2.4; postintervention score mean 16, SD 2.7; *P*<.001), but this was not the case for the PHQ-9 score (preintervention score: mean 11.5, SD 7.5; postintervention score: mean 8, SD 7.0; *P*=.12). Saying that, of these 22 participants, 8 (36%) had a “clinically significant” change of ≥5 points between the start and finish of HeadOn. Of these 22 participants, 7 (32%) had a clinically significant improvement in their PHQ-9 scores, and 1 (5%) had a worsening score. Participants with a complete functional recovery (GOSE score of 8) had statistically significantly lower Rivermead (mean 7, SD 6 vs mean 25, SD 14; *P*=.008) and PHQ-9 (mean 4, SD 3 vs mean 13, SD 8; *P*=.02) scores compared with those who did not have a complete recovery at the end of HeadOn. Of the 29 participants who responded, 10 (34%) represented to a health care professional because of their postconcussion symptoms. Of the 25 participants who were known to be in employment before the concussion, 10 (40%) had returned to work by the end of HeadOn.

#### Postconcussion Symptom Burden and Temporal Profile

A total of 494 symptom diaries were completed by 90% (45/50) of the study participants during the 5-week HeadOn program. On average, 11 entries were completed per participant, with a range of 1 to 34. The symptom diary allowed participants to rate the severity of 8 postconcussion symptoms from 0 (none) to 4 (severe), giving each symptom diary a cumulative range from 0 to 32. Across the 494 completed diaries, the average postconcussion symptom burden was 12 (SD 8). Fatigue, sleep disturbance, and difficulty concentrating were the top 3 symptoms based on overall severity (Figure 3B). As detailed previously, engagement with the symptom diary diminished over time, with a peak of 43 entries on day 1. The average number of entries per day was 14 (SD 7). Daily symptom burdens were highly variable, but the average score diminished over the 5-week period from a peak of 14 (SD 7) in the first week to 7 (SD 5) in the final week (Figure 3C). For the 22 participants with Rivermead scores on completion of HeadOn, there was a strong positive correlation (*r*=0.86; *P*<.001) between their average daily HeadOn symptom diary score and their end-of-program Rivermead score.

## Discussion

### Principal Findings

Concussions are a major public health issue [3]. Although historically viewed as benign, there is a growing understanding that individuals experience a constellation of symptoms and can be left with substantially impaired function [5,50]. This has led to calls for a more systematic and targeted approach to follow up on and support individuals who have sustained a concussion [3]. In reality, many individuals who have experienced a concussion have limited information or follow-up after they are discharged from an ED [10,11]. To address this problem, we developed HeadOn—a digital health intervention specifically designed to support individuals in their concussion recovery. We aimed to incorporate evidence-based educational, behavioral, and psychological interventions that have been found to improve outcomes in concussion [51]. This included early educational material, which Ponsford et al [12] found to significantly reduce postconcussion symptom burden in a randomized controlled trial. We also incorporated elements of CBT, which several trials have demonstrated to have a positive impact on postconcussion symptom burden and recovery. Mittenberg et al [13] examined the effects of an early single session of CBT and found that it significantly reduced the duration of postconcussion symptoms and led to fewer symptoms at the 3-month follow-up. Potter et al [52] examined the delivery of CBT to patients with persistent postconcussion symptoms and found that CBT led to a significant reduction in symptom burden. In addition, we included the introduction of exercise as a recent systematic review found that symptom-limited aerobic exercise has a significant beneficial effect on symptomatic recovery compared with controls [14]. Finally, we also incorporated interventions that have been found to be effective in other diseases and, theoretically, should be effective for concussion, including symptom monitoring [53], alcohol reduction [54], and breathing exercises [55].

HeadOn was developed using a systematic evidence-, theory-, and person-based approach based on the MRC guidance on the development of complex interventions [19]. This approach has been used by others to develop digital health interventions. Bradbury et al [56] developed a digital intervention for cancer survivors. We used a similar approach to that of Bradbury et al [56] but structured the development process to align with the latest MRC guidance, which was recently published [19]. This framework is divided into 4 phases: development of the intervention, feasibility, evaluation, and implementation. In this paper, we describe the first 2 phases—development and feasibility testing. As part of the development process, we synthesized the literature examining what the facilitators of and barriers to using concussion-related interventions were. This information was then used to identify potential barriers to behavior change to address during the behavioral analysis. Some of the key barriers identified during the scoping review were forgetfulness, lack of time, skepticism regarding the ability to fully recover, and lack of motivation. We also identified several contradictory barriers and facilitators, such as minimal in-person contact being both a barrier to and a facilitator of using concussion interventions. This highlights the range of different attitudes in individuals who have experienced a concussion given their demographic breadth and differing recovery trajectories. Importantly, it demonstrates the need to develop a flexible intervention that can fit different use patterns. The BCW was then used as the theoretical model for intervention development [41]. This allowed for mapping of the behavioral analysis onto the BCW and the taxonomy of behavior change techniques [42]. The logic model then provided a framework to conceptualize how HeadOn would work and affect patient outcomes. We then used a person-based approach and conducted optimization studies to understand patient needs and views, which were incorporated into the design to maximize engagement [57]. As recommended in the MRC complex intervention framework, refinement of the intervention should happen after each stage. Therefore, a major component of our mixed methods feasibility study was to obtain feedback from patients who used HeadOn. Quantitative feedback using the MAUQ was broadly very positive, indicating high levels of usability and satisfaction among responders [46]. The MAUQ has been recently introduced and so has not yet gained widespread uptake; however, HeadOn has similar levels of usability compared with other digital interventions in the literature, including those targeting childhood feeding and pediatric burns [58,59]. The qualitative interviews also highlighted some important areas for refinement, including increasing the number of notifications, adding a remove unit function to the alcohol tracker, and altering the language used in the thought monitor. Along with these areas for improvement, difficulty looking at the screen was also mentioned by several interviewees. Concussion guidance is to limit screen time early after an injury. A clinical trial looking at this question found that patients who abstained from screen time for the first 48 hours after their injury had a shorter recovery than those permitted to engage in screen time [60]. Although HeadOn is delivered mostly through a screen (mobile or desktop), there is the option of consuming some of the content through audio. Coupled with this, in the trial, the screen time–abstinent group had 130 minutes of screen time in the first 3 days after their injury. HeadOn can be delivered with limited screen time (5-10 minutes per day), which could easily be achieved within the 130 minutes of the intervention group quoted in the screen time RCT.

Engagement with digital interventions is an important area and, therefore, we aimed to examine how patients engaged with HeadOn during the feasibility study. We found that the initial high levels of engagement trailed off rapidly during the first week. The driving force behind engagement after this was a group of *high engagers* who constituted approximately one-quarter of the cohort (13/50, 26%). Chien et al [61] examined the issue of user engagement in a cohort of 54,604 users of an internet-delivered mental health intervention. In total, 5 categories of engagers were identified: low engagers (36.5%), late engagers (21.4%), high engagers with rapid disengagement (25.5%), high engagers with moderate decrease (6.0%), and highest engagers (10.6%). Even though Chien et al [61] used a more nuanced definition of engagement, the finding of higher engagers, consisting of approximately one-third of the cohort, broadly fits with the findings of this study. One of the key aspects of patient engagement with HeadOn was the completion of the symptom diary. During the feasibility study, a total of 494 diaries were completed. These data provided interesting insights into postconcussion symptom burden and recovery trajectories. Our study found that physical symptoms such as headache and dizziness were some of the least problematic symptoms. This is in contrast to a prospective study by McMahon et al [5], who found physical symptoms to be the most common in a cohort of 348 patients. The same study found a gradual decline in the Rivermead Post-Concussion Symptom Questionnaire score over time between 3 and 6 months. Although we did not follow up on patients for as long as 6 months, we found a gradual decline in their average daily symptom burden over the 5-week duration of HeadOn. Despite the natural history of postconcussion symptoms to decline over time, there is a substantial percentage of patients who are left with persistent postconcussion symptoms. Cnossen et al [4] found that, in a cohort of 591 patients, 41% had persistent postconcussion symptoms at 6 months after their injury. The authors found that the main predictors of developing persistent symptoms included female gender, postconcussion symptoms at 2 weeks, and posttraumatic stress at 2 weeks. One of the key difficulties in implementing predictive models such as this one into routine care is that patients who experience concussion are not commonly followed up on [10,11]. HeadOn addresses this problem by collecting symptom data digitally and could be cost-effectively scaled. These data could then be used to stratify patients and deliver personalized support.

### Study Limitations

This study has several limitations. This includes a low number of participants in the optimization studies during development compared with other authors [56]. We attempted to address this by designing the feasibility study with person-based methodology and conducting qualitative interviews to gain feedback on HeadOn. These interviews, though highly useful, were also prone to bias because of the substantially higher proportion of high engagers. We attempted to reach out to patients who engaged less with the intervention but were unable to receive feedback from them. During recruitment for the feasibility study, the demographic data and the reasons why patients declined participation were not recorded. Therefore, we were unable to determine whether there was recruitment bias in the feasibility study, in particular whether this bias may have contributed to the digital health divide [62]. Finally, our definition of engagement was based on the metric of the patient inputting data into HeadOn. We would have missed patients who accessed the application without inputting data. Some authors argue that these types of basic engagement metrics are too blunt and advocate for the use of measures of cognitive investment to obtain a true impression of engagement with digital interventions [63].

### Conclusions

In this paper, we describe the development of a digital health intervention for concussion using a rigorous evidence-, theory-, and person-based approach. Behavioral theory was used to optimize the intervention to encourage positive behavior change during recovery from concussion. Using the person-based approach, the intervention was optimized through multiple rounds of feedback, which led to participants reporting high levels of usability during the feasibility study. Symptom data input as part of the intervention provided interesting insights into postconcussion symptom burden and, in the future, will provide a means to better target patients at risk of persistent symptoms who require more support. This work lays a robust foundation that supports the progression to the evaluation and implementation phases of the MRC complex intervention development framework.

## Appendix

Multimedia Appendix 1 Supplementary material.

## Abbreviations

BCW: Behavior Change Wheel
CBT: cognitive behavioral therapy
CONSORT: Consolidated Standards of Reporting Trials
COREQ: Consolidated Criteria for Reporting Qualitative Research
ED: emergency department
GOSE: Glasgow Outcome Scale Extended
MAUQ: mHealth App Usability Questionnaire
MRC: Medical Research Council
PHQ-9: Patient Health Questionnaire-9
PRISMA: Preferred Reporting Items for Systematic Reviews and Meta-Analyses

## Data Availability

The clinical feasibility study data are freely available from the University of Edinburgh Datashare [64].
