# Supplementary material for: A Digital Health Intervention for Concussion: Development and Clinical Feasibility Study
Source: JMIR Form Res. 2023 Feb 1;7:e43557. doi: 10.2196/43557 (PMC9932878; doi:10.2196/43557)
Supplement: Multimedia Appendix 1 [file formative_v7i1e43557_app1.pdf]

MULTIMEDIA APPENDIX 1

Article:
d'Offay C, Ng XY, Alexander L, Grant A, Grahamslaw J, Pagliari C, Reed MJ, Carson A, Gillespie DC, Jamjoom AAB: A Digital Health Intervention for Concussion: Development and Clinical Feasibility Study. *JMIR Form Res 2023;7:e43557*

Content	Page
**Tables**	
S1: Barriers and facilitators identified from the scoping review mapped onto 7 domains relevant to HeadOn development	2
S2: Behavioural analysis table	7
S3: Optimisation study 1 feedback results table	12
S4: mHealth App Usability Questionnaire results table	13
S5: Individual interviewee responses of barriers and facilitators mapped onto COM-B framework	15
S6: Qualitative interview feedback (including selected quotations) and associated changes to HeadOn	17
**Figures**	
S1: Flow diagram of HeadOn feasibility study design	20
S2: Flow diagram of HeadOn feasibility study recruitment	21
**Supplementary material**	
S1: Search strategy for scoping review	22
S2: Preferred Reporting Items for Systematic reviews and Meta-Analyses extension for Scoping Reviews (PRISMA-ScR) Checklist	24
S3: Optimisation study 1 feedback questionnaire	27
S4: CONSORT 2010 checklist of information to include when reporting a pilot or feasibility trial	29
S5: Schedule for qualitative interviews	32
S6: Consolidated criteria for reporting qualitative studies (COREQ)	33

Supplementary table 1: Barriers and facilitators identified from the scoping review mapped onto 7 domains relevant to HeadOn development (reference list found in main article)

Intervention characteristics	Facilitators	Barriers
**Intervention engagement**	• Support from others with similar experiences [20-24]<br>• Support networks (eg, return-to-work therapists, friends and family, and the government) [21,25,26]<br>• Lack of social pressure and sense of anonymity as interventions do not involve face-to-face contact [27]<br>• Interpersonal relationship with therapists [21,22,27,28]<br>• Sense of duty or obligation to complete intervention [27]<br>• Self-accountability for recovery [22,29,30]<br>• Self-reflection on condition [21]<br>• Open-minded attitude from the outset [30]<br>• Trusted sources of information/advice at appropriate stage (ie, sports coaches before injury and health care professionals after injury) [26,31]<br>• Belief that pros of the intervention outweigh the cons [30]<br>• Open communication between stakeholders (eg, transparency between patients and employers and notification of schools by health care professionals) [21]<br>• Interventions relieving some stress/responsibility from support networks [32]	• Lack of interaction among intervention participants [20]<br>• Juggling of other commitments leading to lack of time or conflicts in scheduling [27,30]<br>• Nonconcussion-related illness interfering with participation [29]<br>• Inability to access normal social support network (eg, sports teams) [24]<br>• Perceived source credibility [25,26]<br>• Discrepancy in standard of care received for previous instances of concussion [26]<br>• “Bad news” stories from other sources affecting expectations of recovery [25]<br>• Lack of adequate support from support networks (eg, accommodations from employers and government disability grants) [21]<br>• Failure to perceive long-term health consequences [22]<br>• Poor communication between stakeholders leading to lack of accommodation in schools and workplaces [21]<br>• Engagement requiring discipline [30]
**Information**	• Passive discovery of information on popular media rather than actively seeking it out [26]	• Already knowing the information provided [20]<br>• Surface-level information only [27]

	Information about concussions:<br>• Information focusing on symptoms and signs [20,23,29,32-34]<br>• Information on potential risks and complications of concussions [20,23,31]<br>• Typical course of recovery [22,32,34,35]<br>• Methods of prevention [26]<br>• Benefits of education [26]<br>• Information on when to seek medical attention [20,29,33]<br>• Risk factors for persistence of symptoms [22]<br>• Information on concussion interventions:<br>• Sleep and rest [20,22,23,29,31-33]<br>• Return to normal activities [20,22,23,29,31-33,35]<br>• Symptom management techniques and strategies (including adaptive illness behaviors) [22,23,27-29,34-36]<br>• Relaxation strategies [22,23,29,32,36]<br>• Information on nutrition [29]	• Too much repetitive information [37]<br>• Inaccurate/outdated information [26]<br>• Discrepancies in information received from different sources [22,25]<br>• Variations in perceived severity of concussion [26]<br>• Using differing terminologies for concussion [26]<br>• Lacking preinjury education on concussion [26]<br>• Lack of knowledge of available resources of information [26]<br>• Mismatch between experience with concussion and the information provided [25]
**Symptom monitoring**	• Provision of daily planners and concussion symptom scales [23,25,32]<br>• Learning to accept uncertain timelines of symptom recovery [29]<br>• Tracking can alert for the need to escalate care [37]<br>• Feedback from others to gauge progress [25]<br>• Screening by an occupational therapist to facilitate return to work [21]	• Information about symptoms not received early in recovery [23,26]<br>• Placing onus for care on support networks (eg, parents and sports coaches) [26]<br>• Lacking ecological validity when measuring symptoms in a controlled environment [25]
**Lifestyle and behavior**	• Gradual return to activity/sport [20,29]<br>• Ability to plan own solutions to problems [27,38]	• Absence of peer/sports team support [24]<br>• Parental anxiety [24]<br>• Low self-efficacy [26]

	• Time-management skills [27]<br>• Interpersonal skills [27,38]<br>• Reducing smoking and drinking [27]<br>• Regulation of symptom interference [28,38]<br>• Personal finance management skills [27]<br>• Web-based intervention not increasing time on the web more than usual [20]	
**Thoughts and emotions**	• Satisfaction with intervention [20,22,23,27,29-33,35,37-39]<br>• Lower levels of hypervigilance [27,38]<br>• Ability to open up [27]<br>• Lower stress levels [27,30,38]<br>• Desire not to let down therapist [27]<br>• Resolution and acceptance [28,38]<br>• Empowerment and motivation [21,28,30]<br>• Sense of control [28,30]<br>• Reduced fear-avoidance of pain [39]<br>• Improved mood [29,30]<br>• Increased confidence regarding management of concussion [29]<br>• Hope for recovery [29]<br>• Internal belief in resilience and capability [25]<br>• Reassured [31]	• Lazy [27]<br>• Stressed [27]<br>• Skepticism [26,27,30]<br>• Bored during rest [24]<br>• Isolated because of inability to access normal social networks [24,36]<br>• Fear/uncertainty about process of returning to normal activity [24,25,29]<br>• Lack of understanding [26]<br>• Low self-efficacy [26]<br>• Culture of resistance [26]<br>• Self-conscious about the activity [30]<br>• Memory issues/forgetfulness [25]<br>• Anxiety about recovery [25]<br>• Belief that full recovery is unachievable [21,22,25]
**Design**	• Theory-/evidence-based content in keeping with best practices [20,23,26,32]<br>• Ability to talk to a professional [20,27,30,37]<br>• Time-efficient [27,30]<br>• Simplicity [30]<br>• Flexibility [22,27,35]<br>• Safe [38]<br>• Tailored [21,22,28,29,31]	• Lack of tailoring to phase of illness [20,23,27,31]<br>• Lack of tailoring to particular population (eg, military personnel) [34]<br>• Symptoms may interfere with willingness and capability to participate in intervention [30,35]

	• Accessible and convenient [20,23,26-28,33]<br>• Minimal contact/not in person [20,27,30,39]<br>• Different types of educational resources (written/video) to suit participant preference or target certain populations [20,26,32,33]<br>• Interesting/engaging [33,40]<br>• Explicit framework (eg, regular scheduling of appointments) [22,25,27,29,39]<br>• Appropriate frequency of intervention [27]<br>• Low-cost or free [33,35]<br>• Ecological validity of simulated paradigms [40]<br>• Multidisciplinary interventions [21]<br>• Ergonomic adaptations in patients' environment [21]<br>• Involvement of support network within intervention [21,22,32]<br>• Financial incentives for study participation and completion [39,40]	• Lack of face-to-face contact (absence of nonverbal cues and high value placed on personal contact with medical professionals) [27,35]<br>• Varying preference for frequency of intervention sessions [27,36]<br>• Interventions may cause symptom exacerbations [39]<br>• Intensity of intervention not suitable [22,29,31]<br>• Reading burden [34]<br>• Not engaging enough [34]<br>• Less regimented approach can make it harder to adhere to rehabilitation objectives [28]<br>• Dislike of participating in phone calls [27]
**Technical aspects**	• Confidentiality and security of personal information [20,37]<br>• Ability to track participant activity by use patterns [20,33,39]<br>• Ability to set reminders and prompts [37,38,40]<br>• Ability to facilitate a self-referral process [23]<br>• Tangible tools and resources that can be accessed without limits [22,23,30,31,35,36]<br>• Option of printing out resources [34]<br>• Ability to generate automated alerts to clinical team [37]	• Technical issues [33,37,40]<br>• Difficult to use [40]<br>• Disruptions or distractions [30]<br>• Lack of technology confidence or internet access [20,34,35,37]<br>• Geographical, financial, and practical constraints [21,22,31,36]<br>• Lack of trained staff [22]

	• Ability to be implemented without extensive training of staff [32]	

Supplementary table 2: Behavioural analysis table. BCW: Behavioural Change Wheel. BCT: Behaviour Change Taxonomy.

**Negative / Barrier**	**Feature**	**Target Construct BCW**	**Intervention Function BCW**	**Behaviour Change Technique (BCT - using 93 BCT taxonomy v1)**
**Target behaviour: intervention engagement**				
Memory issues / forgetful ness	Information on how to set up a routine around logging in<br><br>Information on setting the web-app to device homepage<br><br>Automated prompts to log-in<br><br>Regular reminders to support behaviour change in the intervention	Psychological capability<br><br>Physical opportunity<br><br>Automatic motivation	Education<br><br>Training<br><br>Persuasion	4.1 Instruction on how to perform a behaviour<br><br>7.1 Prompts and cues<br><br>12.1. Restructuring the physical environment
Credibili ty of source	Meet the team section with description of experts involved in developing the intervention to boost credibility of the intervention	Automatic motivation	Persuasion	9.1 Credible source
Lack of motivati on	Information on the benefits of managing recovery<br><br>'Days in a row' toolbar displays number of consecutive days logged in and days missed.	Reflective motivation<br><br>Automatic motivation	Education<br><br>Incentivisation	5.1 Information about health consequences<br><br>10.4 Social reward
Lack of tailoring to concussi on	Targeted information about concussion recovery	Reflective Motivation	Education	5.1 Information about health consequences
**Target behaviour: Symptom monitoring**				

Information provided is not suitable or contains discrepancies	Accurate information on symptoms and symptom management.<br><br>Advice on when users may need to contact healthcare providers or emergency services.	Psychological capability<br><br>Physical opportunity<br><br>Reflective motivation<br><br>Social opportunity	Education<br><br>Persuasion<br><br>Enablement	5.1 Information about health consequences<br><br>9.1 Credible source<br><br>3.1 Social support (unspecified)
Lack of willingness	Information on the benefits of symptom monitoring<br><br>Allow users to view their symptom diary data<br><br>Ability to record triggers in symptom diary<br><br>Automated reminders to complete diary	Reflective motivation<br><br>Automatic motivation<br><br>Physical opportunity	Enablement<br><br>Persuasion	5.1 Information about health consequences<br><br>1.5 Review of behaviour goal<br><br>4.2 Information about antecedents<br><br>1.4 Action planning<br><br>7.1 Prompts and cues
Lack of time	Flexible monitoring. Ability to input symptom diary daily at any time of day/ place convenient for user<br><br>Automated reminders to complete diary<br><br>Information on habit formation (i.e completing at the same time every day or associating with another activity.	Psychological capability<br><br>Physical opportunity<br><br>Social Opportunity<br><br>Automatic motivation<br><br>Reflective motivation	Education<br><br>Training<br><br>Environmental restructuring<br><br>Enablement<br><br>Incentivisation	4.1 Instructions on how to perform the behaviour<br><br>7.1 Prompts and cues<br><br>1.4 Action planning<br><br>8.3 Habit formation

Lack of skills or ability	Easy navigation and visual system.<br><br>Video instruction on how to use symptom diary	Psychological capability<br><br>Physical capability	Education<br><br>Training<br><br>Enablement	4.1 Instructions on how to perform the behaviour<br><br>6.1 Demonstration of the behaviour
**Target behaviour: Adopt healthy lifestyle habits (good sleep hygiene, increased exercise, reduction in alcohol consumption)**				
Lack of motivation	Information on the benefits of healthy habits in relation to concussion<br><br>Prompts to record their alcohol consumption and automated alert when they have reached their maximum units of consumption for the week.<br><br>Setting goals to perform exercise	Reflective motivation<br><br>Automatic motivation<br><br>Social Opportunity	Education<br><br>Incentivisation<br><br>Restriction	5.1 Information about health consequences<br><br>9.1 Credible source<br><br>7.1 Prompt and cues<br><br>1.4 Action planning<br><br>8.3 Habit formation
Lack of knowledge	Examples of types of exercise<br><br>Information on recommended consumption of units of alcohol and how to count them<br><br>Information on how to form habits. Set regular activity three times per week. Set alcohol free days.<br><br>Instruction on setting up sleep routine and sleep hygiene.	Psychological capability<br><br>Automatic motivation<br><br>Reflective Motivation<br><br>Social opportunity	Education<br><br>Training<br><br>Enablement<br><br>Incentivisation<br><br>Restriction<br><br>Modelling	5.1 Information about health consequences<br><br>1.1 Goal setting (behaviour)<br><br>1.4 Action planning<br><br>4.1 Instructions on how to perform the behaviour<br><br>7.1 Prompts and cues<br><br>8.1

				Behavioural practice/rehearsal<br><br>8.3 Habit formation
Fear or uncertainty about returning to normal activity levels	Information on the benefits of healthy habits in relation to concussion	Psychological capability<br><br>Reflective motivation	Education<br><br>Training<br><br>Persuasion	5.1 Information about health consequences
**Target behaviour: Address negative thoughts**				
Scepticism that a full recovery is unachievable after a concussion	Cognitive restructuring to recognise negative, inaccurate thoughts and replace them with alternative ones that are more realistic and helpful<br><br>Thought monitor to encourage reflection on thoughts about concussion	Psychological capability<br><br>Reflective motivation<br><br>Social opportunity	Education<br><br>Training<br><br>Persuasion<br><br>Enablement	5.6 Information about emotional consequences<br><br>11.2 Reduce negative emotions<br><br>13.2 Reframing
Lack of motivation	Information on how concussion can affect your thoughts.	Psychological capability<br><br>Reflective motivation	Education<br><br>Training	9.1 Credible source<br><br>5.6 Information about emotional consequences
Stress	Breathing exercise to encourage relaxation	Psychological capability<br><br>Physical opportunity	Education<br><br>Training<br><br>Environmental restructuring	12.6 Body changes
**Target behaviour: Goal setting**				
Lack of time	Information and advice on creating goals/ intentions<br><br>Automated reminders about set goals	Psychological capability	Education<br><br>Training	1.1 Goal setting (behaviour)<br><br>1.4 Action planning

				7.1 Prompts and cues
Lack of social support	Advice on sharing goals/intentions with support network<br><br>Review activity goals	Psychological capability<br><br>Reflective motivation<br><br>Social opportunity	Education<br><br>Training<br><br>Environmental restructuring<br><br>Incentivisation	3.1 Social support (unspecified)<br><br>12.2 Restructuring the social environment<br><br>1.5 Review of behaviour goal

Supplementary table 3: optimisation study 1 feedback results table (n=19)

	1 (Poor)	2 (Fair)	3 (Good)	4 (Very Good)	5 (Excellent)
**Overall concept**	0 (0%)	1 (5.3%)	4 (21.1%)	11 (57.9%)	3 (15.8%)
**Symptom diary**	0 (0%)	0 (0%)	9 (47.4%)	7 (36.8%)	3 (15.8%)
**Thought monitor**	0 (0%)	1 (5.3%)	10 (52.6%)	6 (31.6%)	2 (10.5%)
**Symptom support**	0 (0%)	0 (0%)	5 (26.3%)	10 (52.6%)	4 (21.1%)
**Progress report**	0 (0%)	0 (0%)	4 (21.1%)	10 (52.6%)	5 (26.3%)
**Reading material**	0 (0%)	2 (10.5%)	7 (36.8%)	8 (42.1%)	2 (10.5%)
**Breathing exercise**	1 (5.3%)	0 (0%)	7 (36.8%)	8 (42.1%)	3 (15.8%)
**Layout**	0 (0%)	1 (5.3%)	7 (36.8%)	8 (42.1%)	3 (15.8%)
**Overall design**	0 (0%)	1 (5.3%)	7 (36.8%)	9 (47.4%)	2 (10.5%)

Supplementary table 4: response to mHealth App Usability Questionnaire (n=29)

	7 (agree)	6	5	4	3	2	1 (disagree)
**Ease of Use**							
The app was easy to use	15 (51.7%)	8 (27.6%)	3 (10.3%)	1 (34.5%)	2 (69.0%)	0 (0%)	0 (0%)
It was easy for me to learn to use the app	18 (62.1%)	7 (24.1%)	2 (6.9%)	0 (0%)	0 (0%)	2 (6.9%)	0 (0%)
I like the interface of the app	15 (51.7%)	6 (20.7%)	5 (17.4%)	1 (3.5%)	2 (6.9%)	0 (0%)	0 (0%)
The information in the app was well organised, so I could easily find the information I needed	19 (65.5%)	5 (17.2%)	4 (13.8%)	0 (0%)	1 (3.5%)	0 (0%)	0 (0%)
I feel comfortable using this app in social settings	14 (48.3%)	7 (24.1%)	1 (3.5%)	3 (10.3%)	3 (10.3%)	1 (3.5%)	0 (0%)
The amount of time involved in using this app has been fitting for me	15 (51.7%)	6 (20.7%)	3 (10.3%)	3 (10.3%)	1 (3.5%)	1 (3.5%)	0 (0%)
I would use this app again	21 (72.4%)	4 (13.8%)	2 (6.9%)	2 (6.9%)	0 (0%)	0 (0%)	0 (0%)
Overall, I am satisfied with this app	17 (58.6%)	3 (10.3%)	6 (20.7%)	3 (10.3%)	0 (0%)	0 (0%)	0 (0%)
**System information arrangement**							
Whenever I made a mistake using the app, I could recover easily and quickly	8 (28.6%)	6 (21.4%)	8 (28.6%)	3 (10.7%)	0 (0%)	1 (3.6%)	2 (7.1%)
This mHealth app provided an acceptable way to receive healthcare services	15 (51.7%)	7 (24.1%)	3 (10.3%)	3 (10.3%)	0 (0%)	0 (0%)	1 (3.5%)
The app adequately	16 (57.1%)	4 (14.3%)	6 (21.4%)	1 (3.6%)	0 (0%)	0 (0%)	1 (3.6%)

acknowledged and provided information to let me know the progress of my actions							
The navigation was consistent when moving between screens	22 (78.6%)	5 (17.9%)	0 (0%)	0 (0%)	1 (3.6%)	0 (0%)	0 (0%)
The interface of the app allowed me to use all the functions (such as entering information, responding to reminder, viewing information) offered by the app	16 (57.1%)	4 (14.3%)	4 (14.3%)	4 (14.3%)	0 (0%)	0 (0%)	0 (0%)
This app has all the functions and capabilities I expect it to have	15 (53.4%)	4 (14.3%)	6 (21.4%)	2 (7.1%)	0 (0%)	0 (0%)	1 (3.6%)
**Usefulness**							
The app would be useful for my health and well-being	19 (67.9%)	4 (14.3%)	4 (14.3%)	1 (3.6%)	0 (0%)	0 (0%)	0 (0%)
The app improved my access to healthcare services	12 (42.9%)	6 (21.4%)	8 (28.6%)	1 (3.6%)	0 (0%)	0 (0%)	1 (3.6%)
The app helped me manage my health effectively	12 (44.4%)	9 (33.3%)	4 (14.8%)	2 (7.4%)	0 (0%)	0 (0%)	0 (0%)

Supplementary table 5: Individual interviewee responses of barriers and facilitators mapped onto COM-B framework

			Interview														
**Factors of influence**			**1**	**2**	**3**	**4**	**5**	**6**	**7**	**8**	**9**	**10**	**11**	**12**	**13**	**14**	**15**
**Capability**	**Physical**	Difficulty registering due to symptom burden						x	x			x					
		Difficulty with screens / devices due to symptom burden			x			x								x	
		Audio or text files	x	x			x	x		x	x		x	x	x	x	
	**Psychological**	Clear Instructions / Easy to use	x		x	x		x	x	x		x	x		x		x
		Forgetfulness / Memory	x	x	x	x		x	x	x	x	x	x	x	x		
		Difficulty locating and log-in / Not native app	x					x		x		x	x	x			
		Build into routine / Set own reminder		x		x		x	x			x	x	x	x		
		Staggered weekly content / Tasks	x			x		x	x	x				x		x	
**Opportunity**	**Social**	Family / network support	x	x		x		x		x	x		x				
		Work or other commitments							x						x	x	x
	**Physical**	Adding website to device home-screen						x	x						x		
		Alcohol tracker – accidental addition of units	x	x	x	x			x		x	x	x	x			
		Flexibility of digital and self-directed		x	x		x	x	x	x			x	x	x		x
		Prompts and reminders supported engagement	x	x		x	x		x	x	x			x	x		x
		Pandemic increased digital											x	x			

		skills / competence															
Motivation	Reflective	Language used in thought monitor	x	x		x		x		x		x	x		x		
		Being part of an academic study / committing			x	x	x	x	x				x		x	x	
		Ability to review progress / symptom diary	x	x	x			x	x	x		x	x	x			
		Credible source	x	x				x	x	x							
		Belief intervention supported recovery	x										x	x			
	Automatic	Feedback; Days in row progress											x		x		
		Built into routine / Set own reminder		x		x		x	x			x	x	x	x		
		High symptom burden	x		x						x		x	x			
		Increased personalisation/ tailoring			x	x		x						x			
		Low symptom burden (10-14 days symptoms improved and engagement reduced)	x		x	x										x	x
		Repetitive task						x					x		x		

Supplementary table 6: Qualitative interview feedback (including selected quotations) and associated changes to HeadOn

Intervention feedback	Participant quote	Changes to be made to HeadOn
Memory loss and forgetfulness were reported as major symptoms by participants. Participants said that they needed further support to remember to engage with the intervention. Many participants cited being prompted to log-in after receiving the weekly email.	"Try to do it every day, well of course with the concussion as well I've sort of been quite forgetful, that's been part of it as well."<br><br>"Well, when I remember, I try to do it every night, try to set myself some time to remember every night, but sometimes forget, I generally try. I would say 5 times a week I'm managing.'	Increased number of emails and notifications to support memory and enhance engagement
Many participants complained of the sensitivity of the alcohol tracker. This meant that they accidently added units they did not consume. The feature did not have a minus button so participants were unable to correct the mistake. Participants reported being frustrated and annoyed by this.	"Yeah, and with the alcohol tracker, um, it I was expecting a number to come up with the amount of alcohol I was supposed to have consumed today, so a wheel that you would hit, and a number would of come up with the number you had hit, drank that day. And I kept pressing it, looking for it, and it come up that I'd drunk 25 units of alcohol and I'd actually drunk none."	Addition of a minus button to the alcohol tracker.
Participants that built a routine around logging into the intervention, for example at the same time every day or with other medication were more successful at engaging with the intervention every day.	"I usually do it in the afternoon, because what I found, some of the times I missed it, it was the evening, and then we sat down to watch TV and I forgot about it. So mid-to late afternoon, is the easiest time to do it for me."<br>"Really was as part of a routine. I had it on my laptop and would log into my laptop once a day and	Provide information about the benefits of setting a routine.

	as part of that I would checking my emails, doing other things, I would do it as part of that process.”	
Participants that set their own reminders to log-in were more successful at logging in and engaging with the intervention	“Every single day or every second day, the longest period I went without was like 3 days. But I set reminders on my phone now to make sure I do it that day.”	Provide information about the benefits of setting reminders and instruction on how to set a reminder.
Participants who saved the intervention to their device home screen, found logging in easier and were more successful at engaging regularly. Participants who did not found it difficult to remember how to log-in and some had a lengthier process for logging in, for example searching in the email for the link.	“I know it isn’t an app but it was saved to my home screen, so I was able to see it there and I was able to remember to go into it.”	Include an automated add to home-screen button and information on the benefits of saving the intervention to the device home-screen.
Many participants stated that they were unable to remember the incident which they got their injury. They found the thought monitor activity challenging because of this. Whilst the thought monitor was designed for reflection on general concussion and recovery, many participants understood it to focus on the incident.	“think it was the most recent one, you had to like write more, it was more than multiple choice. You had to write out a wee thing about how you were feeling and it was just like the same question, but I couldn’t really write about what had happened because it’s just what people had told me. So I felt a bit useless. I couldn’t fill it out that well.”	Review and edit the language in the thought monitor.
Difficulty with registering and using intervention in first few days after injury, due to high symptom burden. Capacity to use screens and focus reduced. Needed social support to register and use app	“there are people like me who are kind of struggling with computer screens and staying of computer and social media in the immediate period after their head injury, and you know that is a little bit of an issue.”	Streamline the registration process to make it easier to do. Sign post users to the audio clips to allow alternative means of consuming HeadOn information without the need to look at a screen

	"Accept that in the first few days I couldn't look at a screen and had difficulty reading and wasn't a very easy format to engage with… so it would have been better if they sent it to a partner and they could of said 'do you want to sign up for this?' You know they know my name. I think it would have been more effective to send it to someone."	

Supplementary figure 1: flow diagram of HeadOn feasibility study design

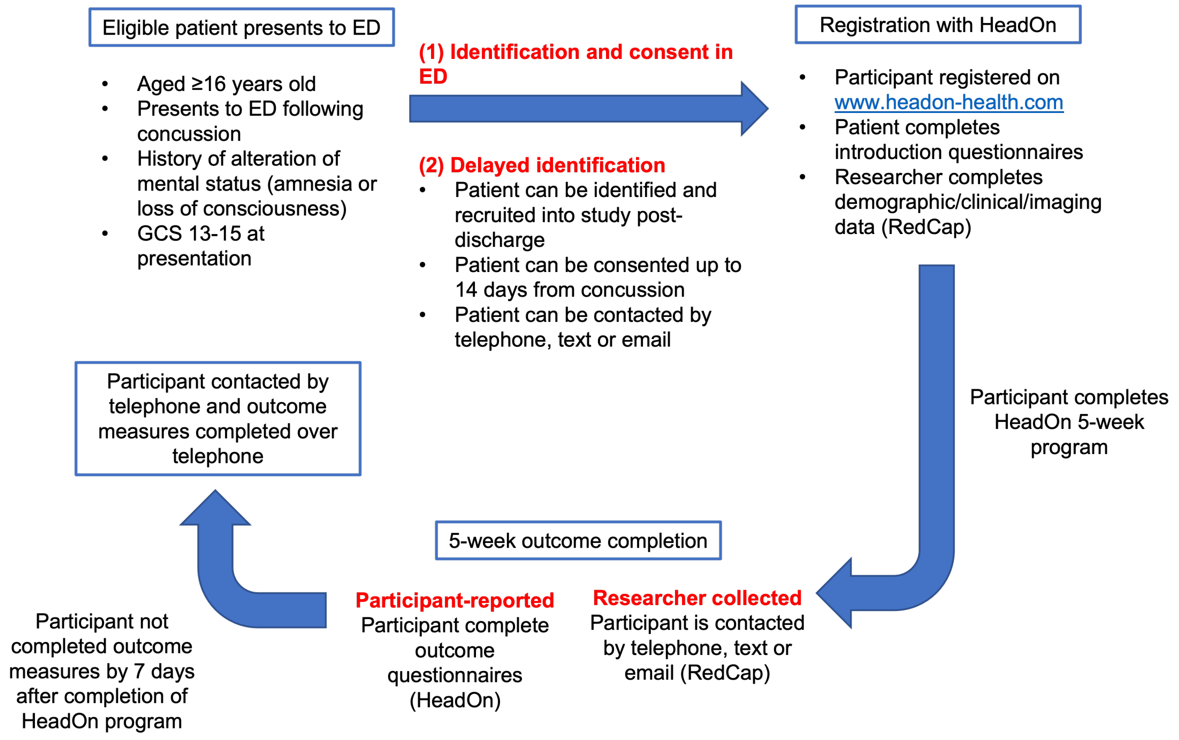

Supplementary figure 2: flow diagram of HeadOn feasibility study recruitment

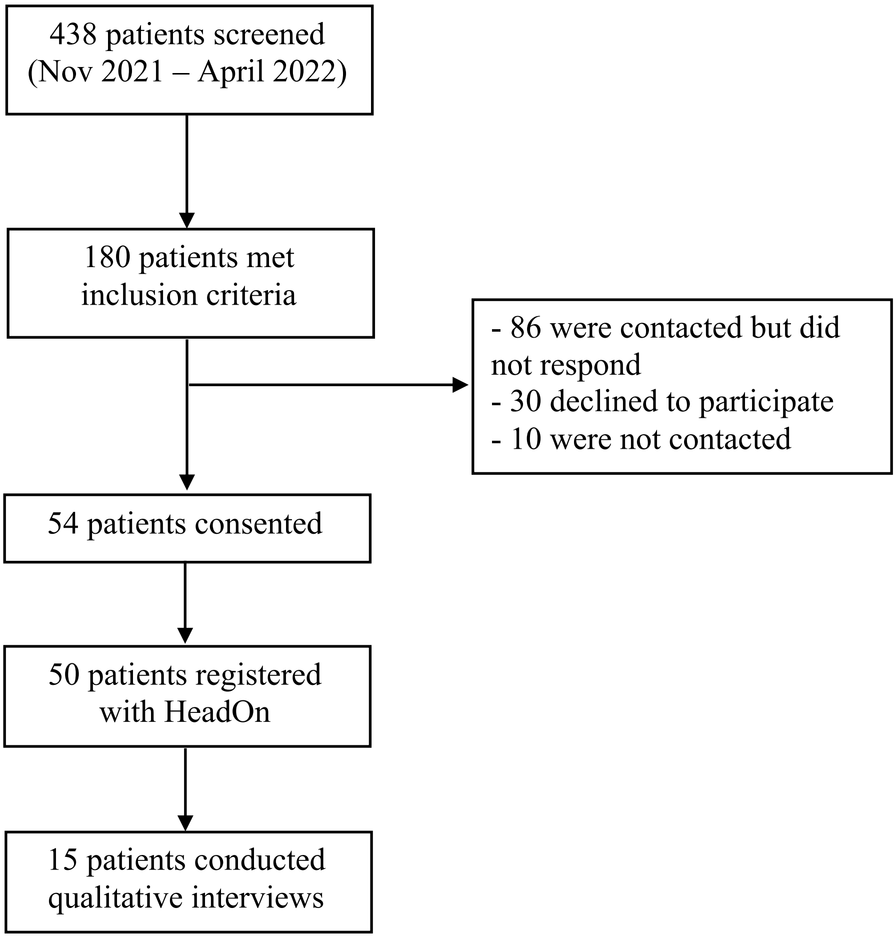

Supplementary material 1: search strategy for scoping review

Database	Search strategy	Hits
MEDLINE(Ovid) and Cochrane Library (Wiley)	1) exp Brain Concussion/rh, th [Rehabilitation, Therapy]<br>2) exp Post-Concussion Syndrome/rh, th [Rehabilitation, Therapy]<br>3) (mild traumatic brain injury or mTBI or mild head injury).ti,ab,id.<br>4) exp Craniocerebral Trauma/rh, th [Rehabilitation, Therapy]<br>5) 1 or 2 or 3 or 4<br>6) exp Self-Management/ or exp Self Care/<br>7) exp Exercise Therapy/ or exp "Physical and Rehabilitation Medicine"/<br>8) exp Health Education/ or exp Patient Education as Topic/ or exp Consumer Health information/<br>9) (psychoeducation or intervention).ti,ab,id.<br>10) exp Behavior Therapy/ or exp Cognitive Behavioral Therapy/ or exp Counseling/mt [Methods] or exp Psychotherapy/th [Therapy]<br>11) exp Bed Rest/<br>12) 6 or 7 or 8 or 9 or 10 or 11<br>13) (("semi-structured" or semistructured or unstructured or informal or "in-depth" or indepth or "face-to-face" or structured or guide) adj3 (interview* or discussion* or questionnaire*)).ti,ab.<br>14) exp Interviews as Topic/mt, px [Methods, Psychology] or exp Focus Groups/mt [Methods] or exp Narration/ or exp Qualitative Research/<br>15) 13 or 14<br>16) 5 and 12 and 15	MEDLINE: 113<br>Cochrane: 109
PsycInfo (APA)	1) (post-concussi* or postconcussi*).ti,ab.<br>2) (mild traumatic brain injury or mTBI or mild head injury).ti,ab.<br>3) brain concussion/<br>4) 1 or 2 or 3<br>5) self-management/ or self care/<br>6) rehabilitation/ or physical therapy/ or exercise/ or movement therapy/ or "rest"<br>7) health education/ or health knowledge/ or health literacy/ or health promotion/ or psychoeducation/<br>8) psychotherapy/ or psychotherapeutic counselling/ or psychotherapeutic	35

	techniques/ or strategic therapy/ or online therapy<br>9) cognitive behavior therapy/ or cognitive rehabilitation/ or neuropsychological rehabilitation/ or ("behavio* therapy" or "cognitive therapy").ti,ab.<br>10) 5 or 6 or 7 or 8 or 9<br>11) (("semi-structured" or semistructured or unstructured or informal or "in-depth" or indepth or "face-to-face" or structured or guide or guides) adj3 (interview* or discussion* or questionnaire*)).ti,ab,id.<br>12) (focus group* or qualitative or ethnograph* or fieldwork or "field work" or "key informant")).ti,ab,id.<br>13) qualitative research/ or interviews/ or group discussion/ or qualitative study.md. or experiences.tw. or interview.tw. or qualitative.tw.<br>14) 11 or 12 or 13<br>15) 4 and 10 and 14	
Scopus	1) TITLE-ABS(post-concussi* or postconcussi* or "mild traumatic brain injury" or mTBI or "mild head injury" or "concussion")<br>2) TITLE-ABS-KEY ("self*management" OR "self*care" OR exercise OR "exercise therap*" OR "physical therap*" OR rehabilitation OR intervention OR "health education" OR "patient education" OR psychoeducation OR "cognitive behavi* therapy" OR cbt OR "behavi* therap*" OR "cognitive therap*" OR "counsel*" OR "rest")<br>3) TITLE-ABS-KEY ("semi-structured" OR semistructured OR unstructured OR informal OR "in-depth" OR indepth OR "face-to-face" OR structured OR guide OR guides OR interview* OR discussion* OR questionnaire* OR "focus group*" OR qualitative OR ethnograph* OR fieldwork OR "field work" OR "key informant")<br>4) #1 and #2 and #3	731

Supplementary material 2: Preferred Reporting Items for Systematic reviews and Meta-Analyses extension for Scoping Reviews (PRISMA-ScR) Checklist

SECTION	ITEM	PRISMA-ScR CHECKLIST ITEM	REPORTED ON PAGE #
**TITLE**			
Title	1	Identify the report as a scoping review.	Page 2
**ABSTRACT**			
Structured summary	2	Provide a structured summary that includes (as applicable): background, objectives, eligibility criteria, sources of evidence, charting methods, results, and conclusions that relate to the review questions and objectives.	n/a the ScR was small part of larger body of work
**INTRODUCTION**			
Rationale	3	Describe the rationale for the review in the context of what is already known. Explain why the review questions/objectives lend themselves to a scoping review approach.	Page 4
Objectives	4	Provide an explicit statement of the questions and objectives being addressed with reference to their key elements (e.g., population or participants, concepts, and context) or other relevant key elements used to conceptualize the review questions and/or objectives.	Pages 4 and 6
**METHODS**			
Protocol and registration	5	Indicate whether a review protocol exists; state if and where it can be accessed (e.g., a Web address); and if available, provide registration information, including the registration number.	As sub-study protocol not registered
Eligibility criteria	6	Specify characteristics of the sources of evidence used as eligibility criteria (e.g., years considered, language, and publication status), and provide a rationale.	Page 6
Information sources*	7	Describe all information sources in the search (e.g., databases with dates of coverage and contact with authors to identify additional sources), as well as the date the most recent search was executed.	Page 6
Search	8	Present the full electronic search strategy for at least 1 database, including any limits used, such that it could be repeated.	Page 6

SECTION	ITEM	PRISMA-ScR CHECKLIST ITEM	REPORTED ON PAGE #
Selection of sources of evidence†	9	State the process for selecting sources of evidence (i.e., screening and eligibility) included in the scoping review.	Page 6
Data charting process‡	10	Describe the methods of charting data from the included sources of evidence (e.g., calibrated forms or forms that have been tested by the team before their use, and whether data charting was done independently or in duplicate) and any processes for obtaining and confirming data from investigators.	na
Data items	11	List and define all variables for which data were sought and any assumptions and simplifications made.	Page 6
Critical appraisal of individual sources of evidence§	12	If done, provide a rationale for conducting a critical appraisal of included sources of evidence; describe the methods used and how this information was used in any data synthesis (if appropriate).	na
Synthesis of results	13	Describe the methods of handling and summarizing the data that were charted.	Page 6
**RESULTS**			
Selection of sources of evidence	14	Give numbers of sources of evidence screened, assessed for eligibility, and included in the review, with reasons for exclusions at each stage, ideally using a flow diagram.	Figure 1
Characteristics of sources of evidence	15	For each source of evidence, present characteristics for which data were charted and provide the citations.	Table 1
Critical appraisal within sources of evidence	16	If done, present data on critical appraisal of included sources of evidence (see item 12).	na
Results of individual sources of evidence	17	For each included source of evidence, present the relevant data that were charted that relate to the review questions and objectives.	na
Synthesis of results	18	Summarize and/or present the charting results as they relate to the review questions and objectives.	Table 1
**DISCUSSION**			
Summary of evidence	19	Summarize the main results (including an overview of concepts, themes, and types of evidence available), link to the review questions and objectives, and consider the relevance to key groups.	Page 29

SECTION	ITEM	PRISMA-ScR CHECKLIST ITEM	REPORTED ON PAGE #
Limitations	20	Discuss the limitations of the scoping review process.	As part of broader piece of research limitations not discussed
Conclusions	21	Provide a general interpretation of the results with respect to the review questions and objectives, as well as potential implications and/or next steps.	Page 32
**FUNDING**			
Funding	22	Describe sources of funding for the included sources of evidence, as well as sources of funding for the scoping review. Describe the role of the funders of the scoping review.	Page 33

Supplementary material 3: optimisation study 1 feedback questionnaire

HEADON FEEDBACK QUESTIONNAIRE

We would like your views to help us develop a new mobile application to help people manage their symptoms after a head injury. This survey will only take you a few minutes and any additional comments you would like to make would be greatly appreciated. Thank you for taking time to complete this survey.

1. **Have you used mobile apps to manage your health?**

   Yes □ No □

2. **Please rate the following app features:**

	1 (Poor)	2 (Fair)	3 (Good)	4 (Very Good)	5 (Excellent)
Symptom diary					
Thought matrix					
Symptom support					
Progress report					
Reading material					
Breathing exercise					
Layout					
Overall design					

3. **Would you be willing to input information about your symptoms if you were to use an app like this?**

   Yes □ No □ Unsure □

4. **How useful do think the app is for the purpose intended?**

   Not useful at all □ Not so useful □ Somewhat useful □
   Very useful □ Extremely useful □

5. **What length of programme would you be able to complete if you were to use an app like this?**

   1 week □ 2 weeks □ 3 weeks □ 4 weeks □

6. **How much would you be willing to pay for this app?**

7. **Would you use this app?**

   Yes ☐ No ☐

8. **Would you recommend this app to other people who have had a head injury?**

   Definitely not ☐ Probably not ☐ Don't know ☐
   Probably ☐ Definitely ☐

9. **Do you have any further comments about the app, or suggestions as to how it could be improved?**

Thank you for taking the time to complete this survey

Supplementary material 4: CONSORT 2010 checklist of information to include when reporting a pilot or feasibility trial

Section/Topic	Item No	Checklist item	Reported on page No
**Title and abstract**			
	1a	Identification as a pilot or feasibility randomised trial in the title	1
	1b	Structured summary of pilot trial design, methods, results, and conclusions (for specific guidance see CONSORT abstract extension for pilot trials)	2
**Introduction**			
Background and objectives	2a	Scientific background and explanation of rationale for future definitive trial, and reasons for randomised pilot trial	4/5
	2b	Specific objectives or research questions for pilot trial	4/5
**Methods**			
Trial design	3a	Description of pilot trial design (such as parallel, factorial) including allocation ratio	8/9
	3b	Important changes to methods after pilot trial commencement (such as eligibility criteria), with reasons	n/a
Participants	4a	Eligibility criteria for participants	8
	4b	Settings and locations where the data were collected	8/9
	4c	How participants were identified and consented	8
Interventions	5	The interventions for each group with sufficient details to allow replication, including how and when they were actually administered	8
Outcomes	6a	Completely defined prespecified assessments or measurements to address each pilot trial objective specified in 2b, including how and when they were assessed	8/9
	6b	Any changes to pilot trial assessments or measurements after the pilot trial commenced, with reasons	n/a
	6c	If applicable, prespecified criteria used to judge whether, or how, to proceed with future definitive trial	n/a
Sample size	7a	Rationale for numbers in the pilot trial	n/a
	7b	When applicable, explanation of any interim analyses and stopping guidelines	n/a
Randomisation:			
Sequence generation	8a	Method used to generate the random allocation sequence	n/a
	8b	Type of randomisation(s); details of any restriction (such as blocking and block size)	n/a
Allocation concealment mechanism	9	Mechanism used to implement the random allocation sequence (such as sequentially numbered containers), describing any steps taken to conceal the sequence until interventions were assigned	n/a

Implementation	10	Who generated the random allocation sequence, who enrolled participants, and who assigned participants to interventions	n/a
Blinding	11a	If done, who was blinded after assignment to interventions (for example, participants, care providers, those assessing outcomes) and how	n/a
	11b	If relevant, description of the similarity of interventions	n/a
Statistical methods	12	Methods used to address each pilot trial objective whether qualitative or quantitative	This was a mixed methods study
**Results**			
Participant flow (a diagram is strongly recommended)	13a	For each group, the numbers of participants who were approached and/or assessed for eligibility, randomly assigned, received intended treatment, and were assessed for each objective	Supplementary material 16
	13b	For each group, losses and exclusions after randomisation, together with reasons	Supplementary material 16
Recruitment	14a	Dates defining the periods of recruitment and follow-up	8
	14b	Why the pilot trial ended or was stopped	8
Baseline data	15	A table showing baseline demographic and clinical characteristics for each group	20/21
Numbers analysed	16	For each objective, number of participants (denominator) included in each analysis. If relevant, these numbers should be by randomised group	21/22
Outcomes and estimation	17	For each objective, results including expressions of uncertainty (such as 95% confidence interval) for any estimates. If relevant, these results should be by randomised group	21/22
Ancillary analyses	18	Results of any other analyses performed that could be used to inform the future definitive trial	n/a
Harms	19	All important harms or unintended effects in each group (for specific guidance see CONSORT for harms)	20
	19a	If relevant, other important unintended consequences	n/a
**Discussion**			
Limitations	20	Pilot trial limitations, addressing sources of potential bias and remaining uncertainty about feasibility	31/32
Generalisability	21	Generalisability (applicability) of pilot trial methods and findings to future definitive trial and other studies	32
Interpretation	22	Interpretation consistent with pilot trial objectives and findings, balancing potential benefits and harms, and considering other relevant evidence	29/30

	22a	Implications for progression from pilot to future definitive trial, including any proposed amendments	31/32
**Other information**			
Registration	23	Registration number for pilot trial and name of trial registry	9
Protocol	24	Where the pilot trial protocol can be accessed, if available	9
Funding	25	Sources of funding and other support (such as supply of drugs), role of funders	33
	26	Ethical approval or approval by research review committee, confirmed with reference number	8

Supplementary material 5: schedule for qualitative interviews

Interview schedule

The researcher will be introduced as, a student from the University of Edinburgh, interested in their views and experiences of using the intervention. To open the interview participants will be initially asked about their concussion and or/use of health apps. The list of topics asked to participants includes;

1. Would you tell me a little about your concussion? Where/when it happened?
2. What impact on your daily life, if any, did your concussion have?
3. What was your experience of using the HeadOn Web application?
4. If you didn't use the intervention, why not?
    a. Did they use any other services /seek out any information from other sources?
    b. Is there anything that may have helped or encouraged you to use
    c. If your symptoms hadn't improved would you have used the intervention
5. If you did use the intervention, when and how they used the intervention
6. how easy or not easy they found the intervention to use
7. likes and dislikes about the intervention
8. the functions/features were most useful and least useful?
9. what changes they would make within the intervention?

Supplementary material 6: Consolidated criteria for reporting qualitative studies (COREQ)

No. Item	Guide questions/description	Reported on Page #
**Domain 1: Research team and reflexivity**		
Personal Characteristics		
1. Inter viewer/facilitator	Which author/s conducted the interview or focus group?	Page 9
2. Credentials	What were the researcher's credentials? E.g. PhD, MD	Page 9
3. Occupation	What was their occupation at the time of the study?	Page 9
4. Gender	Was the researcher male or female?	Page 9
5. Experience and training	What experience or training did the researcher have?	Page 9
Relationship with participants		
6. Relationship established	Was a relationship established prior to study commencement?	Page 9
7. Participant knowledge of the interviewer	What did the participants know about the researcher? e.g. personal goals, reasons for doing the research	Page 9
8. Interviewer characteristics	What characteristics were reported about the interviewer/facilitator? e.g. Bias, assumptions, reasons and interests in the research topic	Page 9
**Domain 2: study design**		
Theoretical framework		
9. Methodological orientation and Theory	What methodological orientation was stated to underpin the study? e.g. grounded theory, discourse analysis, ethnography, phenomenology, content analysis	Page 10
Participant selection		
10. Sampling	How were participants selected? e.g. purposive, convenience, consecutive, snowball	Page 9
11. Method of approach	How were participants approached? e.g. face-to-face, telephone, mail, email	Page 9
12. Sample size	How many participants were in the study?	Page 9 and Table 4
13. Non-participation	How many people refused to participate or dropped out? Reasons?	Supplementary figure 2

Setting		
14. Setting of data collection	Where was the data collected? e.g. home, clinic, workplace	Page 9
15. Presence of non-participants	Was anyone else present besides the participants and researchers?	No – page 9
16. Description of sample	What are the important characteristics of the sample? e.g. demographic data, date	Table 4
Data collection		
17. Interview guide	Were questions, prompts, guides provided by the authors? Was it pilot tested?	Supplementary material 4
18. Repeat interviews	Were repeat inter views carried out? If yes, how many?	No
19. Audio/visual recording	Did the research use audio or visual recording to collect the data?	Yes – page 9
20. Field notes	Were field notes made during and/or after the interview or focus group?	n/a
21. Duration	What was the duration of the interviews or focus group?	Page 9
22. Data saturation	Was data saturation discussed?	Yes - Page 10
23. Transcripts returned	Were transcripts returned to participants for comment and/or correction?	No
**Domain 3: analysis and findings**		
Data analysis		
24. Number of data coders	How many data coders coded the data?	One - CD
25. Description of the coding tree	Did authors provide a description of the coding tree?	n/a
26. Derivation of themes	Were themes identified in advance or derived from the data?	Derived from data and mapped onto pre-defined domains – page 21
27. Software	What software, if applicable, was used to manage the data?	NVivo – page 10
28. Participant checking	Did participants provide feedback on the findings?	No
Reporting		
29. Quotations presented	Were participant quotations presented to illustrate the themes/findings? Was each quotation identified? e.g. participant number	Supplementary table 5

30. Data and findings consistent	Was there consistency between the data presented and the findings?	Yes
31. Clarity of major themes	Were major themes clearly presented in the findings?	Table 5
32. Clarity of minor themes	Is there a description of diverse cases or discussion of minor themes?	Page 23/24
